# The impact of a cash transfer programme on tuberculosis treatment success rate: a quasi-experimental study in Brazil

**DOI:** 10.1136/bmjgh-2018-001029

**Published:** 2019-01-24

**Authors:** Daniel J Carter, Rhian Daniel, Ana W Torrens, Mauro N Sanchez, Ethel Leonor N Maciel, Patricia Bartholomay, Draurio C Barreira, Davide Rasella, Mauricio L Barreto, Laura C Rodrigues, Delia Boccia

**Affiliations:** 1 Department of Infectious Disease Epidemiology, London School of Hygiene and Tropical Medicine, London, UK; 2 Department of Medical Statistics, London School of Hygiene and Tropical Medicine, London, UK; 3 Tropical Medicine Department, University of Brasília, Brasília, Brazil; 4 Federal University of Brasília, Brasília, Brazil; 5 Federal University of Espírito Santo, Vitoria, Brazil; 6 National Tuberculosis Programme/Ministry of Health, Brasília, Brazil; 7 National Tuberculosis Programme/Ministry of Health of Brazil, Brasília, Brazil; 8 Centro de Pesquisas Gonçalo Muniz, Fundação Oswaldo Cruz, Salvador, Brazil; 9 Institute of Collective Health, Federal University of Bahia, Salvador, Brazil; 10 Centro de Integração de Dados de Conhecimentos para Saúde (CIDACS), Fundação Oswaldo Cruz, Salvador, Brazil

**Keywords:** tuberculosis, social protection, conditional cash transfer, Bolsa Família, propensity score matching, quasi-experimental design, causal inference

## Abstract

**Background:**

Evidence suggests that social protection policies such as Brazil’s Bolsa Família Programme (BFP), a governmental conditional cash transfer, may play a role in tuberculosis (TB) elimination. However, study limitations hamper conclusions. This paper uses a quasi-experimental approach to more rigorously evaluate the effect of BFP on TB treatment success rate.

**Methods:**

Propensity scores were estimated from a complete-case logistic regression using covariates from a linked data set, including the Brazil’s TB notification system (SINAN), linked to the national registry of those in poverty (CadUnico) and the BFP payroll.

**Results:**

The average effect of treatment on the treated was estimated as the difference in TB treatment success rate between matched groups (ie, the control and exposed patients, n=2167). Patients with TB receiving BFP showed a treatment success rate of 10.58 percentage points higher (95% CI 4.39 to 16.77) than patients with TB not receiving BFP. This association was robust to sensitivity analyses.

**Conclusions:**

This study further confirms a positive relationship between the provision of conditional cash transfers and TB treatment success rate. Further research is needed to understand how to enhance access to social protection so to optimise public health impact.

**Key questions**

**What is already known?**

- While encouraging, evidence about the impact of cash transfers on tuberculosis (TB) control is still scattered and conclusions are often hampered by important study limitations.

**What are the new findings?**

- This is the first study using a quasi-experimental design to evaluate the impact of Bolsa Familia on TB treatment success.
- Patients with TB enrolled in Bolsa Familia are more likely to complete their treatment successfully.
- Approximately half of patients with TB included in this study population were not enrolled in the cash transfer programme despite being eligible based on the income inclusion criterion.

**What do the new findings imply?**

- Conditional cash transfers like Bolsa Familia can contribute to TB elimination even if they were not designed for this purpose.
- Disparity in access is a missed opportunity to maximise TB impact of Bolsa Familia.

## Introduction

Despite biomedical efforts, the global burden of tuberculosis (TB) remains considerable, with up to 1.5 million deaths from TB recorded in 2015.^1^ TB treatment takes many months, and a proportion of patients are not cured, either because they abandon treatment, take treatment irregularly, are infected with drug-resistant TB, or die before completion of treatment.^1^ The correlation between TB indicators and global poverty has been demonstrated both at ecological and individual levels, yet much of the morbidity and mortality in patients with TB still occur among the poorest segments of the population.^2^ Social determinants impact vulnerability to TB at every stage of the disease pathway, from TB infection to clinical outcomes, including whether or not the patient was successfully treated.^3^ Ending the global burden of TB requires bold policies and supportive systems able to recognise and tackle these social determinants.^4^

Recognising this social aspect of TB epidemiology, social protection is now a non-negotiable component to reach the TB elimination targets set by the WHO, including zero households affected by catastrophic costs, defined as TB-related expenditures when they exceed 20% of preillness annual household income.^5^ Brazil in particular has been an early adopter of the WHO’s End TB Strategy,^6^ as reflected by its long-term efforts to integrate development and health agendas. This is partially due to the long social protection tradition in Latin America, which in Brazil culminated with the creation of the Bolsa Família Programme (BFP) in 2003, one of the largest conditional cash transfer programmes in the world.^7^

In 2010, the BFP provided a variable monthly stipend to households meeting certain socioeconomic criteria: households earning less than R$70 a month (~US$22 at time of writing) and households with children, adolescents or pregnant women earning less than R$‎140 a month. BFP’s targeting is not exact, and individuals reporting an income above R$‎140 can be found in the BFP payroll.^7^ In order to receive BFP, families must be registered in the Cadastro Unico (single registry; CadÚnico), a registry of all low-income Brazilian families. In return for the transfers, recipients must comply with behavioural obligations (ie, school attendance; immunisation). BFP is not explicitly intended to target TB-affected households and only one-fourth of patients with TB in Brazil appear to be enrolled in the programme; given the intimate association between poverty and TB, underenrolment is likely.^8^ Despite accumulating, the literature on the impact of conditional cash transfers on a variety of TB indicators is still limited, and there has been little methodologically rigorous evaluation of social protection interventions for TB prevention, care and control, including treatment outcomes.^9^ There has also been some support in the literature for financial incentives having a small positive effect on TB outcomes,^10^ but the underlying philosophy, mechanisms of action, as well as the ethical and sustainability implications for financial incentives may differ from cash transfers embedded into proper governmental social protection platforms.^11^

Despite its scarcity, the evidence is converging on a consistent positive impact of social protection on TB epidemiology and control, including some small-scale trials and studies in Peru,^12^ Moldova^13^ and South Africa.^14–16^As for Brazil, the literature is even more rich even if evidence does not necessarily follow from proper controlled trials.^15–18^ Torrens *et al* 8 have already attempted to estimate the impact of BFP on TB treatment success rates and found out that patients with TB enrolled in BFP were approximately 7% more likely to be successfully treated after treatment than a control group.^8^ While the findings of this study are consistent with what observed in the literature, conclusions are hampered by the potential biased nature of the control group.^8^

For an unbiased estimate of the proportion of patients cured attributable to BFP, we must construct a control group as similar as possible to the group of BFP recipients. This group of BFP recipients on average have some TB treatment success rate. We wish to estimate the difference in that treatment success rate if, counter to fact, that group of patients had not received BFP, but had the same sociodemographic characteristics and were thus still enrolled in CadÚnico.

To this aim, we approach the same routine data source as in Torrens *et al* 8 using a quasi-experimental approach to construct a more appropriate control group and to then determine a more rigorous estimate of the effect of BFP on TB treatment success rate among those who receive it. Specifically, we aimed to: (1) use propensity score matching to create a control group balanced for propensity to receive BFP, (2) provide an estimate of the average treatment effect of BFP on TB treatment success rate among recipients and (3) to reflect on the utility of the resulting estimate for changing TB policy.

## Methods

### Conceptual framework: directed acyclic graph

**Figure 1:**
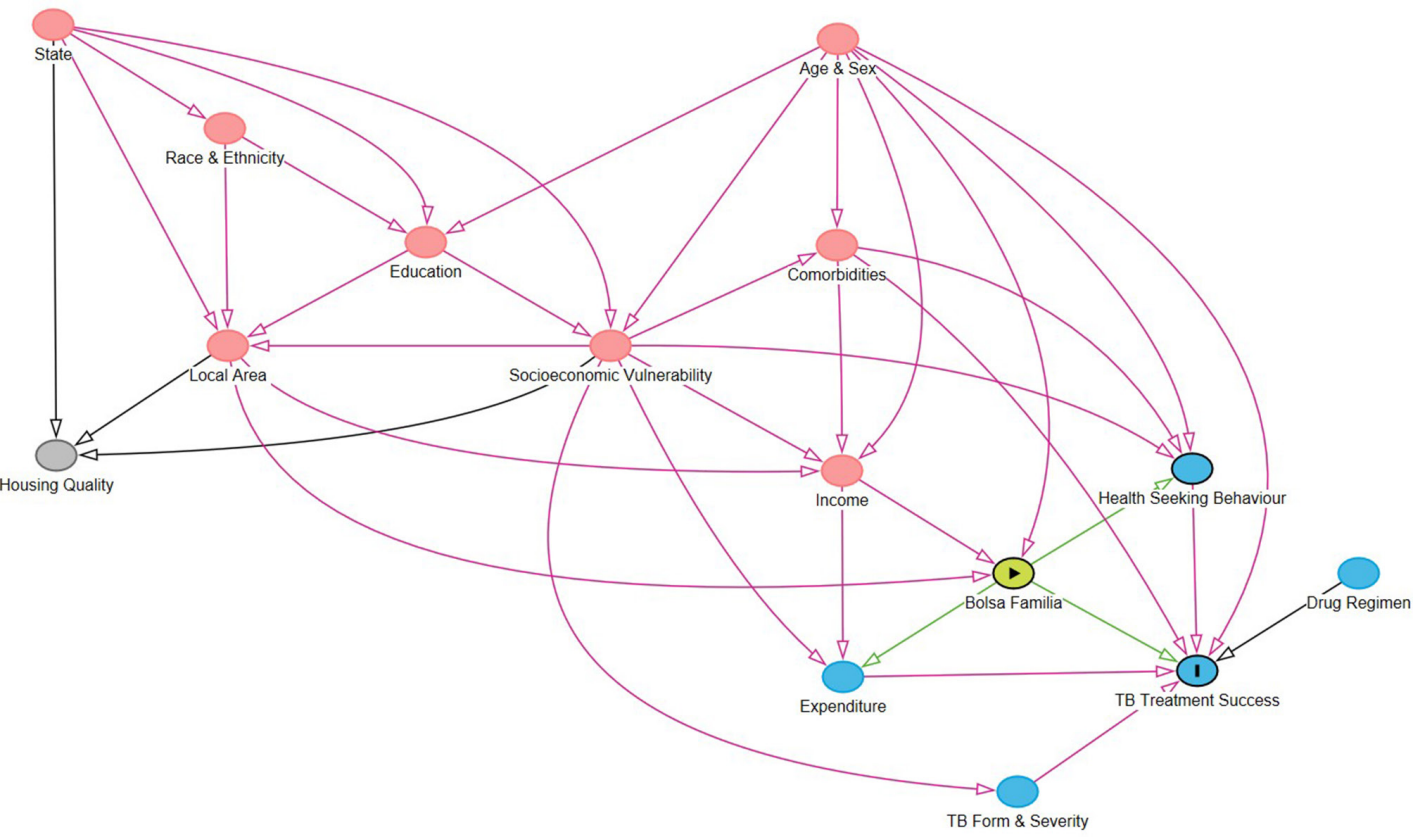
Directed acyclic graph (DAG) outlining the pathways linking Bolsa Familia with tuberculosis (TB) outcomes. A DAG was built to conceptualise the potentially causal relationships between constructs relevant for measuring the impact of Bolsa Familia on TB treatment success rate. Red nodes are ancestors of both the outcome and the exposure (ie, confounders) while grey nodes are unassociated with the outcome and exposure. Blue nodes are ancestors of the outcome. The DAG links nodes that represent constructs that are measured by covariates table 2).

A directed acyclic graph (DAG) was proposed for conceiving of the causal relationships between the outcome, the exposure and all the variables hypothesised to be on the causal pathway (figure 1). Each node in the DAG consists of a high-level construct measured by proxy variables taken from the set of covariates available (table 1). The nodes in this DAG were constructed based on a variety of theoretical literature, and the grouping of covariates under one node denotes that they are considered to be measures of that underlying construct for the purposes of this paper.^3 19 20^ Online supplementary appendix 1 outlines explicitly which covariates fall under each node.

10.1136/bmjgh-2018-001029.supp1 Supplementary data

**Table 1:** Variables to operationalise constructs included in the statistical models

Node (construct)	Covariates included in the model	Covariates excluded from the model (missing data threshold)	Covariates excluded from the model (no available measure)
State	State		
Race	Race, indigenous, quilombola		
Local area	Urbanicity, running water, sewage, electricity, water store, garbage collection	House type	Transit access
Education	Years of education, literacy		
Socioeconomic vulnerability	Child work, institutionalisation, work-acquired TB	Employment, pension receipt, unemployment benefit, alimony receipt	Food security, adequate nutrition, perception of poverty
Age and sex	Age, sex		Gender identity
Comorbidities	AIDS, alcohol use disorder, diabetes, HIV, mental disorder, other chronic illness		General mental health, stress
Income	Income		
Expenditure	(on) Food, energy, gas, water	(on) Rent, transport	Medical costs
Health-seeking behaviour	Directly observed treatment		Engagement with primary care
TB form and severity	Chest X-ray, initial sputum smear, pulmonary/extrapulmonary, throat culture, tuberculin skin test		MDR-TB (is included in outcome as non-successful treatment)
Drug regimen	Rifampicin, isoniazid, ethambutol, streptomycin, pyrazinamide, ethionamide, other drugs		

Not all covariates included under one of the constructs in the directed acyclic graph (DAG) were included in the propensity score model. Table 1 summarises which covariates were included and which were excluded. Some covariates that might reasonably be part of the pathways encoded in this DAG were excluded as there was no adequate measure of them in these linked administrative data. Other covariates were excluded by the missing data threshold, which itself was chosen to balance measurability of each of the constructs with the loss of sample size from undertaking a complete case analysis.

The housing quality node was not included in the model as it was not associated with outcome (TB mortality) or exposure. The housing node included measurable covariates of roof, floor, and wall material, number of people in the home, and the number of bedrooms and bathrooms, as well as the unmeasurable covariate of indoor air pollution.

MDR-TB, multidrug-resistant tuberculosis; TB, tuberculosis.

**Table 2:** Results of propensity score matching estimates of the ATT for four models

Models* n controls=898 n exposed=1269	ATT	95% CI	Controls matched (unweighted), n	Exposed dropped, n	Pairs matched (weighted), n	Unique controls, n
Model A†	10.58	(4.39 to 16.77)	6021	109	1160	545
Model B‡	7.21	(1.33 to 13.09)	6468	21	1248	656 (D2)

Models* n controls=1319 n exposed=1729	ATT	95% CI	Controls matched (unweighted), n	Exposed dropped, n	Pairs matched (weighted), n	Unique controls, n
Model C*	6.31	(1.46 to 11.16)	8895	70	1659	955
Model D*‡	7.06	(2.57 to 11.56)	9272	17	1712	1001

The matching used was many-to-one with replacement. Some exposed patients were not similar enough to any control patients according to the calliper threshold and these individuals were dropped from the analysis (exposed dropped). Some controls were not similar enough to any exposed patients and were thus not used as potential matches and dropped from the analysis. The remaining controls (unique controls) were then ‘copied’ a number of times to be used as potential matches (controls matched unweighted). Each control was not matched individually, but rather weighted to form one matched comparator for each treatment patient. These matched comparator patients were matched to the treatment patients to form matched pairs (pairs of controls and treated cases matched). The number of pairs may thus be higher than the total initial sample size as some controls were used more than once and some were not used at all.

*Models C and D omit variables with >25% missing data.

†Model A includes linear and quadratic forms of continuous covariates and omits variables with >50% missing data to estimate the propensity score. Variables included in the final propensity score are those listed in bold in the caption to figure 1.

‡Models B and D omit quadratic forms of continuous covariates.

ATT, average effect of treatment on the treated.

The DAG outlines potential mechanisms by which BFP (‘the exposure’) is proposed to affect treatment success rate (‘the outcome’). These include via access to directly observed treatment and via increased capacity for mitigation of catastrophic costs (expenditure). We provide an estimate for the direct effect of social protection outside of these pathways, which may include expanded access to healthcare through means other than Directly Observed Therapy (DOT), increased psychological well-being or greater integration into governmental systems in general. The DAG also outlines pathways between treatment success rate and income (and therefore access to BFP), through complex relationships between demographics, geography and socioeconomic factors. The ‘treatment success’ outcome includes those who completed treatment with or without bacteriological confirmation.

### Data handling

The data for this study arose from a linkage between the 2010 TB data set from SINAN (Brazil's national Notifiable Disease Surveillance System) and the 2011 CadÚnico data set. The CadÚnico data set was itself linked to the Bolsa Familia payroll held by the Caixa Federal (Federal Bank). The linkage added the demographic and social information from CadÚnico and the BFP payroll to every patient with TB in the SINAN data set.

Of the complete SINAN-CadÚnico-BFP data set (n=180 046), only individuals who were new TB cases registered in CadUnico in 2010 with a non-missing treatment outcome variable were retained for this study (n=16 760). Exposed individuals (defined here as those receiving BFP) were further restricted to those whose receipt of BFP preceded case closure. Case closure is defined as the date on which an outcome (eg, treated, unsuccessful completion of treatment, death) is recorded. The final data set used for analysis included 13 029 individuals, 6940 of whom received BFP. The data set contained a set of 60 covariates that could be used for propensity score matching (ie, categorical or numerical data).

Many of these 60 covariates had a considerable amount of missing data. Data were assumed to be missing completely at random. Variables that were recorded as missing in over 50% of individuals were omitted from the analysis. These variables included house type (permanent/improvised), roof, floor, and wall material, number of people and families in the home, number of bedrooms and bathrooms, variables relating to employment status, expenditure on rent and transport, and receipt of pension, unemployment benefit and alimony. It is conceivable that rent and transport expenditure could be important confounders of treatment success rate given the potential of cash transfers for mitigating catastrophic costs, but neither are conditionally associated with both outcome and exposure in the observed data and expenditure is represented by other retained variables.^21^

The omission of variables with this level of missing data resulted in 45 covariates to be considered for use in propensity score estimation. A sensitivity analysis was run omitting all variables with over 25% missing data, which further omitted water expenditure and years of formal education. At both missing data thresholds, at least one proxy covariate remained under each node of the DAG such that no high-level construct was unrepresented by the available covariates.

### Propensity score matching

Without applying propensity score approaches or other approaches to control for confounding, it is likely that the values of the available covariates between the exposed and the unexposed (and those who experience or do not experience the outcome) vary, which potentially biases comparisons between groups. We wish to achieve a ‘balance’ in these values, which may approximate the balance produced by conventional randomisation procedures. We wish to first determine the likelihood of receiving BFP given the covariate values, which is represented by the propensity score. If the propensity score is then balanced between groups by matching, it is as though the covariates that were used to estimate the propensity score were themselves balanced.^22^

Propensity scores were estimated by logistic regression. One of two criteria must be met for a variable to be included in this logistic regression: (A) conditional association with the outcome given exposure, to improve precision or (B) both association with exposure and conditional association with outcome given exposure, to account for confounding.^23^ These criteria apply to both mediators and confounders and can be determined from the DAG (figure 1). All DAG nodes meet these criteria but housing and thus the covariates used to model the propensity score were all non-housing covariates meeting the missing data threshold. Quadratic forms of the continuous covariates were used in the logistic regression but sensitivity analyses were performed without including them. Two-way interactions between gender and all variables and age and all variables were also used, given it is likely that these covariates would differ in effect across strata.

Each patient who did not receive BFP (ie, not exposed) was matched to a patient who did receive it (ie, exposed) closest in propensity score, within a particular ‘caliper' of 0.1 SD from the mean propensity score. Matching was done with replacement and multiple matches to minimise both bias and variance, following Caliendo and Kopeinig.^24^ Multiple matches were weighted to form one matched control for each patient. Standardised mean differences and overlap plots were examined to assess whether balance was improved by matching.

Throughout the literature, complete cases are used for propensity score matching, and this is the approach used in this paper.^24^ This reduced the data set to 2167 individuals at the 50% missing data threshold and 3048 individuals at the 25% threshold.

### Estimating the impact of Bolsa Familia

Taking the difference of the proportion of treatment success between matched groups resulted in an estimate of the average effect of treatment on the treated (ATT), or the (causal) risk difference in the exposed. The procedure used in Abadie and Imbens^25^ was used to estimate the SE of the ATT and thus the CIs. The CIs thus account for the uncertainty due to the matching procedure, but do not account for the uncertainty due to the fact that the estimated propensity score is itself a function of the data; this latter feature leads to conservative inferences.^25^ The ATT was also estimated by a multiple imputation-based sensitivity analysis, and point estimates from this are provided for comparative purposes in online supplementary appendix 2.

### Statistical software

All analyses were conducted in R V.3.4.1 and the MatchIt package was used for the propensity score matching procedure.

## Results

### Propensity score matching: covariate balance

**Figure 2:**
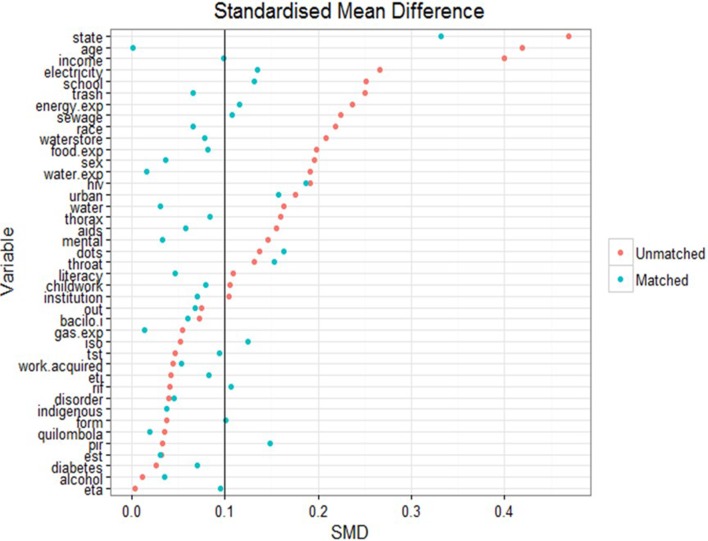
Standardised mean difference (SMD). The change in SMD in the matched and unmatched groups for each variable. A smaller difference indicates improved balance between groups; being below the threshold of 0.1 is conservatively considered to be effectively balanced. Balance has been largely improved by matching though some imbalance remains between groups. bacilo.i, initial sputum smear; disorder, any other chronic illness; est, streptomycin; eta, ethambutol; eti, ethionamide; exp, expenditure; iso, isoniazid; ﻿mental, mental disorder; pir, pyrazinamide; rif, rifampicin; thorax, chest X-ray; throat, throat culture; tst, tuberculin skin test.

**Figure 3:**
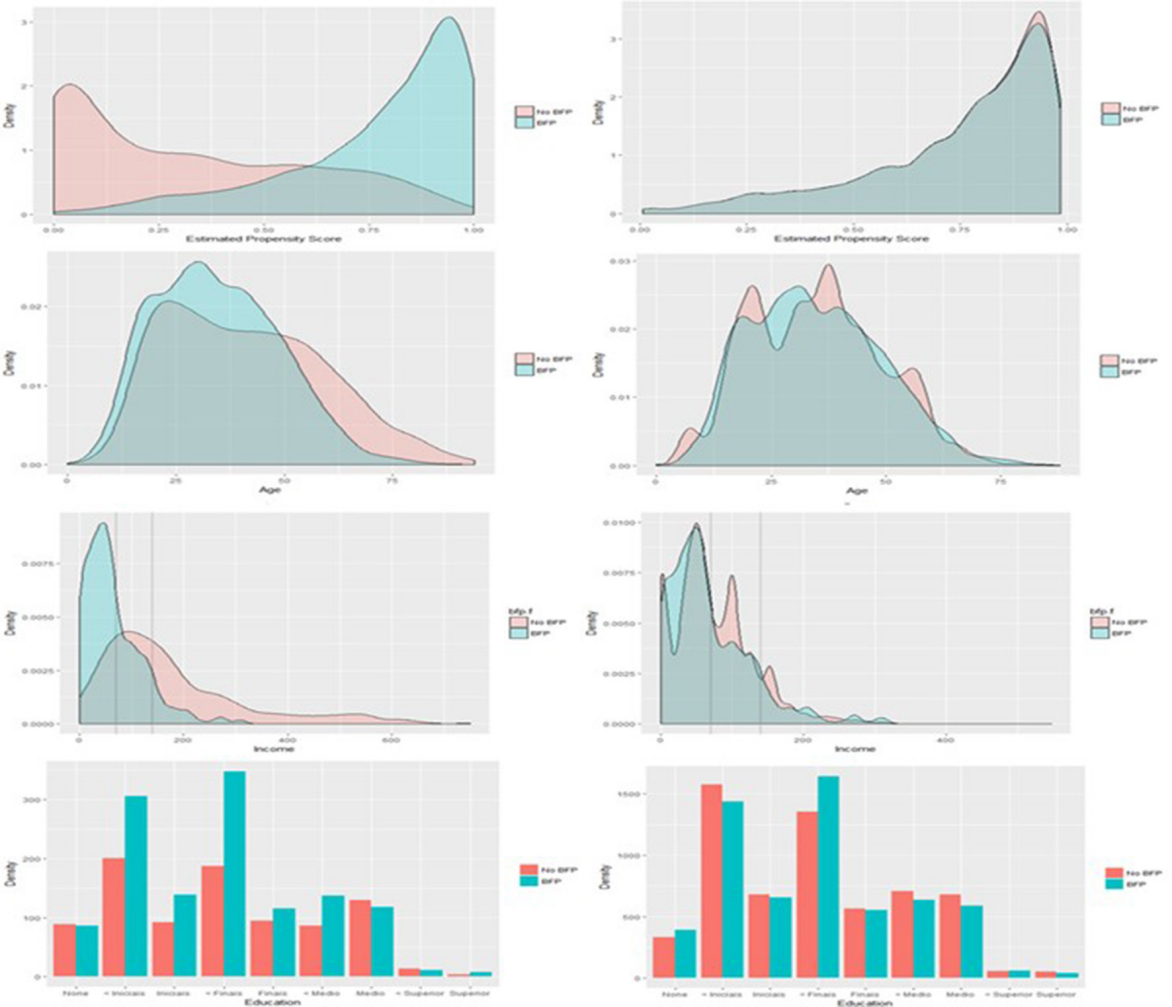
Overlap in estimated propensity scores between those receiving and those not receiving Bolsa Família Programme (BFP) before matching (top left) and after matching (top right). Overlap has been substantially improved by matching to treated (exposed) patients, suggestive of the groups being balanced on the propensity score. The region of overlap extends between 0 and 1. Also presented are similar plots of variable distribution before and after matching for income, age and schooling (from top to bottom). Dotted lines on the income distributions mark the thresholds for BFP eligibility.

A complete balance table is presented in table 1 in online supplementary appendix 1 for the match produced by model A for all covariates included in the propensity score matching exercise. There is good similarity of the covariates after matching, suggesting a reasonable balance was obtained between groups. Prior to matching, there were some imbalances found between BFP recipients and non-recipients on important covariates. Figure 2 presents the changes in standardised mean difference between those receiving BFP (ie, exposed) and those not receiving BFP (ie, not exposed) before and after matching. Figure 3 presents overlap plots to demonstrate the similarity of the propensity score values between groups.

Propensity score matching in general resulted in improved balance of the values of covariates between cases and controls. A standardised mean difference of below 0.1 implies that groups do not differ greatly between values of the covariate.^23^ Though the matching process only brought 50% of the imbalanced variables below this threshold, a large improvement was seen on the balance of important upstream covariates like age (0.42 to 0.01), income (0.40 to 0.09) and schooling (0.24 to 0.12). The change in distributions of these variables after matching can be seen in figure 3. On average, those receiving BFP in the unmatched cohort were younger (34.5 vs 41.3 years), poorer (R$65.2 vs R$197.4 per month) and less educated (89.2% vs 83.5% not completed secondary school).

From figure 3, up 20.9% of patients with TB fall under the R$70 income threshold for unconditional receipt of BFP and therefore are theoretically eligible for the programme, but yet excluded from it. A further 29.4% fall under the R$140 income threshold and could therefore potentially be eligible for BFP.

### Estimating the impact of Bolsa Familia

In total, four estimates of the ATT were produced (table 2). Model A is the primary model of interest as it is the most complex model specification. Models B–D represent sensitivity analyses on model A to investigate how sensitive the results are to simplifying changes to these modelling and missing data decisions.

The ATT from model A was estimated to be 10.58 (95% CI 4.39 to 16.77) (table 2). Thus, among patients with TB who receive BFP, we expect a treatment success rate of 10.58 percentage points higher than if those patients had not received the benefit. The proportion successfully treated in those who did not receive BFP was 76.6% compared with 87.2% in the BFP recipients. This average treatment effect is protective even when a simpler model is used and when the missing data threshold at which covariates are omitted is reduced to 25%, with ATT estimates between 6.31 and 7.21 (table 2). It is also in broad agreement with an ATT point estimate of 7.22 obtained from a multiple imputation approach (online supplementary appendix 2). Expressed as number needed to treat, the estimated ATT implies that on average, among patients with TB who received Bolsa Familia before acquiring TB, one unsuccessful treatment outcome was averted because of Bolsa Familia for every nine patients.

## Discussion

### Summary: interpretation of results

This is the first study that uses a quasi-experimental approach to estimate the impact of a conditional cash transfer programme on TB treatment success rates.^9^ Across all models, results have shown a substantial absolute increase in TB treatment success rate (between 7% and 11%) among those who receive BFP. This seems to suggest a consistent positive association between receiving BFP on a key indicator of TB control: treatment success rate. This is in line with the studies of Torrens *et al* 8 and Durovni *et al* 15 and a few other previous studies evaluating the relationship between social protection and TB outcomes undertaken using less rigorous methodologies and adjusting for only a subset of potential confounders, which also demonstrate a protective effect of similar scale.^13 26^ Given the already relatively high treatment success rate in Brazil, it can be expected that the size of impact may be even higher in settings within and outside Brazil, with lower treatment success rates and a less effective TB control programme. Similar propensity score approaches have already been used to evaluate the effect of cash transfers in HIV/AIDS, but not on TB.^27^

Another important and somewhat unexpected finding of our analysis is that the profile of patients with TB enrolled in BFP was not overtly dissimilar from patients with TB who have not received BFP even before matching. Figure 2 suggests that the most imbalanced covariates for receipt of BFP (based on the standardised mean difference) were state of residence, income, age and schooling. There may also be differences between recipients and non-recipients based on measures of the infrastructure of the local area (sewage, electricity, trash disposal). Patients with TB not benefiting from BFP transfers appear to be broadly similar to patients with TB who are BFP recipients under a number of other sociodemographic characteristics, particularly on comorbidities such as diabetes and alcohol abuse, as well as on DOT prevalence table 2 in online supplementary appendix 1. This suggests there may be some shared vulnerability among patients with TB (ie, concomitant socioeconomic stressors, diverse ability to navigate complex social services), who are not captured by the current BFP targeting and enrolment process, leading to some degree of disparity in access to social protection and specifically BFP in Brazil. Even when looking strictly to the BFP eligibility criterion (ie, income), our results show that up to 51.3% of patients may be theoretically eligible for BFP, but yet left out. This seems to further suggest that the income threshold for BFP is insufficiently specific to ensure access to vulnerable patients with TB.

## Strength and limitations

The utilisation of quasi-experimental approach is a major strength of this paper. Quasi-experimental approaches like propensity score matching require fewer assumptions about the data than traditional parametric counterparts. The specification of the estimand and population parameters of interest are an additional strength to using propensity score matching, and the risk of bias from residual confounding is minimised compared with prior work by careful use of a DAG.^28^ While the use of propensity scores for matching has recently drawn some criticism,^29^ the diagnostic plots demonstrated in figures 2 and 3 show that balance was improved by matching, and a number of model specifications for the propensity score were tested and found to demonstrate a similar positive impact.

Indeed, a clear strength of this work is the comparability of the control group. As demonstrated in figure 3, those in the exposed group and those in the control group have a very similar distribution of propensity to receive BFP. This overlap suggests that we are only comparing patients with similar covariate profiles: while some of the control patients may not be eligible on paper for BFP, in the complex context of real-world receipt of BFP, the not-exposed group (our ‘control’ group) resemble almost exactly those patients with TB who receive BFP on all measured variables and are representative of a broad range of patients with TB from across Brazil. This is a methodological improvement over the control groups seen in prior work which greatly strengthens the quality of evidence available to policymakers.

The control group in the study of Durovni *et al* 15 was taken from a pool of all patients with TB rather than those who are registered in CadÚnico, and therefore some patients ineligible for BFP were included in the control group. The control group in the study of Torrens *et al* 8 was taken from patients with TB who were eligible in theory for BFP, but who had not received any money from the programme until after treatment. This control group had characteristics different from those patients with TB not eligible for the programme on demographic and socioeconomic variables examined by the authors. Both of these control groups may have potentially biased the resulting estimate of proportion of patients cured attributable to BFP.

This quasi-experimental approach also implies the possibility of drawing causal conclusions. The estimand used in this study, the average treatment effect on the treated, could be given a causal interpretation if particular ‘identifying’ assumptions hold, including: (1) positivity, which implies that no individual has a probability of 1 of receiving BFP conditional on their confounders; (2) consistency, which implies that different variations of receiving BFP do not have different effects on TB outcomes; and (3) conditional exchangeability, which implies that there is no residual confounding. We note that while BFP might appear to create a structural violation of the positivity assumption with its income threshold, examining the threshold itself it was noted that the cut-off was often inaccurately applied and thus very few random positivity violations were encountered in the matched set. With regard to the consistency assumption, we specifically assumed that receipt of any amount of transfer for any amount of time was sufficient in this context, but further work should investigate dose–response relationships between cash transfers and TB. Drawing causal conclusions is however hampered by the non-interference assumption, which in this context assumes that the exposure received by one individual does not affect the outcome of the other. The results of this study suggest that the size of effect found may be too large to ignore this assumption and work should be undertaken to investigate the effect of social protection on TB transmission. Another potential violation of this assumption is that BFP increases the probability of treatment success in recipients and in other cases through community effects of the cash transfer.

In conclusion, while most identifying assumptions are potentially plausible, we cannot draw conclusions about causality given the interference limitations outlined above. The circumstances under which causal inferences can be drawn with interference is an area of ongoing research.^30^

Another limitation to this work is the data quality. The missing data results in a relatively small sample size used for matching and we cannot rule out the possibility of residual confounding from covariates that are mostly missing or remain unbalanced. Remaining imbalance on the state variable suggests data may be missing conditionally at random on the state variable. As information on it is housed within a separate register, we were unable to assess the impact of the Family Health Strategy, (FHS) though previous work suggests the effect of BFP is independent of Family Health Strategy (FHS) coverage.^15^ While an approach combining multiple imputation and propensity score matching would have mitigated this problem, there remain many gaps in the literature on the practical implementation of these techniques together (see online supplementary appendix 2). Furthermore, the data linkage is cross-sectional and thus time-varying confounding cannot be accounted for with these data; better data availability longitudinally would allow for measurement on more direct measures of TB control, such as incidence.

The choice of a dichotomous outcome variable may be another limitation: non-success outcomes include continued disease after regimen completion, treatment abandonment, death from TB, death from other causes and development of multidrug-resistant TB, which may have heterogeneous risk factors. Loss to follow-up and transferred cases are also not considered by this analysis—the analysis is agnostic about whether these patients were cured or not cured. The results may be different if each non-success outcome were addressed in turn, but this would require a larger sample size and may be best addressed in a descriptive study.

### Policy implications

Despite the above limitations, these findings preliminarily suggest that: (1) there is a considerable proportion of patients with TB eligible for BFP that for unknown reasons seem to be left out from the programme; (2) almost half of the patients with TB will not be eligible for BFP according to income thresholds, and thus there is room for a more comprehensive or multidimensional targeting approach not only using income as eligibility criteria. Given the 7%–11% absolute increase in treatment success rate seen among those receiving BFP from our work, from a health rights perspective, it must be considered how best to deliver a protective programme to vulnerable patients in Brazil.

BFP was not designed to address specific diseases, not least TB: TB status is not a targeting criterion and none of the conditionalities currently imposed by the programme have any direct implication for TB care and/or TB control. Despite the suggested positive impact, ethical and equity issues make unlikely that TB will become one of the eligibility criteria of BFP. Nonetheless, access could be expanded, and thus impact maximised, by making BFP more TB sensitive through a more inclusive, although non-stigmatising, targeting strategy. Higher impact could in fact be achieved by simply ensuring that patients who are already eligible by definition for the programme receive the benefits, or at least receive them while on treatment. To this purpose, further research is urgently needed to understand determinants of access to BFP from patients with TB and to explore those supply and demand side barriers that delay the transfer of benefits once patients with TB are legitimately enrolled.

Understanding how to effectively and cost-effectively remove these individual and system-level barriers and what may be the ultimate impact on the Brazilian TB epidemic is a priority research area, whose lessons may be transferrable to other settings.

Nonetheless, it can be anticipated that the removal of these barriers may require the implementation of more efficient BFP delivery models, including the ‘single window’ approach which entails an integrated delivery of TB care services and social protection.^31^ According to this model, the access to the most appropriate social protection schemes is determined and facilitated at the primary healthcare level where ad hoc staff (eg, social workers) are trained to assess the social protection needs of patients with TB and provide information, legal and administrative advices, and referrals to various services so to allow patients to access benefits from one ‘single window’ without having to navigate across complex and multiple service points.^31^

Another emerging model for the delivery of social protection is the ‘cash plus’ model in which the provision of cash transfers is combined with another form of social support when the provision of in-kind benefits is not deemed sufficient to reduce households’ vulnerabilities (including health-related vulnerabilities).^32^

In the case of TB in Brazil, this ‘plus’ component can be represented by a top-up of the cash benefit to account for the TB-related catastrophic costs incurred by the households; or the provision of a food basket to improve nutrition of cash beneficiaries and therefore their treatment outcome; or the improvement of housing and ventilation conditions to interrupt intrahousehold transmission of TB. To identify the most relevant ‘intensifier’ of any cash transfer intervention it will be essential also to understand thoroughly the most likely pathway through which this impact takes place. This requires the development of a setting-specific, epidemiologically driven conceptual framework and a more comprehensive collection of data for the variables in the causal pathway.

To be useful the above research agenda should rely on both quantitative and qualitative methods to embrace the complexity of pathways likely to underlie impact and the multifaced nature of determinants of access to cash transfers in the context of TB-affected communities.

## Conclusions

Overall, the strength of evidence and size of effect of the ATT estimated in this work seems to suggest that expanding social protection to a wider population of patients with TB may represent a valid mechanism for improving TB outcomes beyond the traditional biomedical approach. This is consistent with the need of a multisectoral accountability framework expressed during the last WHO-Global Ministerial Conference held in Moscow in November 2017 which demands a more pervasive integration of TB programmatic action within development models and infrastructures.^33^ It is essential that, like in this work, recent developments in quasi-experimental methodology continue to be integrated with the evidence base for bold policies in development. With stronger evidence available, the rapid implementation of bold policies may be justified in TB contexts and the global public health community will be a large step closer to achieving the aims of the WHO’s End TB Strategy.
