# Supplementary material for: The impact of a cash transfer programme on tuberculosis treatment success rate: a quasi-experimental study in Brazil
Source: BMJ Glob Health. 2019 Jan 24;4(1):e001029. doi: 10.1136/bmjgh-2018-001029 (PMC6347926; doi:10.1136/bmjgh-2018-001029)
Supplement: Supplementary data [file bmjgh-2018-001029supp001.pdf]

Appendix 1 - Baseline Characteristics.

The following table presents the baseline characteristics of the unmatched population and the same characteristics after matching. Variables correspond to the covariates of the DAG in Figure 1 in the manuscript that were included in the propensity score model.

Table 1. Balance on covariates both before and after matching, stratified by receipt of Bolsa Familia.

	Unmatched		*Matched (unweighted)*	
**Group**	**No BFP**	**BFP**	**No BFP**	**BFP**
N	898	1269	6021	6021
**Sex** (%)				
F	418 (46.5)	711 (56.0)	3432 (57.0)	3323 (55.2)
I	0 (0.0)	1 (0.1)	0 (0.0)	0 (0.0)
M	480 (53.5)	557 (43.9)	2589 (43.0)	2698 (44.8)
**Age** (mean (sd))	41.28 (18.19)	34.45 (14.21)	34.86 (14.26)	34.85 (14.20)
**Race** (%)				
Branca	311 (34.6)	321 (25.3)	1430 (23.8)	1550 (25.7)
Preta	122 (13.6)	199 (15.7)	1081 (18.0)	965 (16.0)
Amarela	10 (1.1)	7 (0.6)	26 (0.4)	36 (0.6)
Parda	451 (50.2)	736 (58.0)	3484 (57.9)	3470 (57.6)
Indigena	4 (0.4)	6 (0.5)	0 (0.0)	0 (0.0)

**Indigenous** = Not Indigenous (%)	896 (99.8)	1268 (99.9)	6007 (99.8)	6016 (99.9)
**Quilombola** = Not Quilombola (%)	895 (99.7)	1267 (99.8)	6012 (99.9)	6016 (99.9)
**Years of Education** (n years)				
None	89 (9.9)	86 (6.8)	333 (5.5)	394 (6.5)
Fundamental I incomplete (< 5)	201 (22.4)	306 (24.1)	1578 (26.2)	1439 (23.9)
Fundamental I complete (5)	92 (10.2)	139 (11.0)	680 (11.3)	657 (10.9)
Fundamental II incomplete (< 9)	187 (20.8)	348 (27.4)	1357 (22.5)	1645 (27.3)
Fundamental II complete (9)	95 (10.6)	116 (9.1)	568 (9.4)	555 (9.2)
Medio incomplete (< 12)	86 (9.6)	137 (10.8)	710 (11.8)	638 (10.6)
Medio complete (12)	130 (14.5)	118 (9.3)	681 (11.3)	590 (9.8)
Superior incomplete (< 16)	14 (1.6)	11 (0.9)	59 (1.0)	60 (1.0)
Superior complete (16)	4 (0.4)	8 (0.6)	55 (0.9)	43 (0.7)
**Literacy** = Illiterate (%)	142 (15.8)	153 (12.1)	632 (10.5)	720 (12.0)
**Urban** (%)				
Urban	838 (93.3)	1121 (88.3)	5064 (84.1)	5383 (89.4)
Rural	54 (6.0)	129 (10.2)	801 (13.3)	544 (9.0)
Periurban	6 (0.7)	19 (1.5)	156 (2.6)	94 (1.6)
**Water** = No Running Water (%)	76 (8.5)	172 (13.6)	800 (13.3)	738 (12.3)
**Sewage** (%)				
Sewage System	461 (51.3)	575 (45.3)	2705 (44.9)	2849 (47.3)
Septic Tank	176 (19.6)	238 (18.8)	1077 (17.9)	1103 (18.3)

Tank	192 (21.4)	372 (29.3)	1953 (32.4)	1717 (28.5)
Open Air	49 (5.5)	59 (4.6)	246 (4.1)	274 (4.6)
Into Water	1 (0.1)	10 (0.8)	2 (0.0)	10 (0.2)
Other	19 (2.1)	15 (1.2)	38 (0.6)	68 (1.1)
**Electricity** (%)				
Own Metered	792 (88.2)	1002 (79.0)	4801 (79.7)	4839 (80.4)
Central Metered	33 (3.7)	64 (5.0)	375 (6.2)	304 (5.0)
Unmetered	53 (5.9)	134 (10.6)	448 (7.4)	591 (9.8)
Gas or Oil	2 (0.2)	9 (0.7)	0 (0.0)	0 (0.0)
Candle	4 (0.4)	8 (0.6)	24 (0.4)	37 (0.6)
Other	14 (1.6)	52 (4.1)	373 (6.2)	250 (4.2)
**Water Source** (%)				
Pipe Network	801 (89.2)	1040 (82.0)	5032 (83.6)	5067 (84.2)
Well	64 (7.1)	154 (12.1)	657 (10.9)	648 (10.8)
Cistern	7 (0.8)	14 (1.1)	101 (1.7)	50 (0.8)
Other	26 (2.9)	61 (4.8)	231 (3.8)	256 (4.3)
**Trash** (%)				
Direct Collect	759 (84.5)	1003 (79.0)	4923 (81.8)	4856 (80.7)
Indirect Collect	14 (1.6)	54 (4.3)	204 (3.4)	223 (3.7)
Household	48 (5.3)	127 (10.0)	547 (9.1)	511 (8.5)
Street	18 (2.0)	22 (1.7)	76 (1.3)	110 (1.8)

Other	59 (6.6)	63 (5.0)	271 (4.5)	321 (5.3)
**Thorax X-Ray** (%)				
Suspect	763 (85.0)	1009 (79.5)	4954 (82.3)	4821 (80.1)
Normal	43 (4.8)	70 (5.5)	286 (4.8)	321 (5.3)
Other Pathology	11 (1.2)	14 (1.1)	26 (0.4)	62 (1.0)
Not Undertaken	81 (9.0)	176 (13.9)	755 (12.5)	817 (13.6)
**Initial Bacilloscopy** (%)				
Positive	206 (22.9)	329 (25.9)	1452 (24.1)	1543 (25.6)
Negative	291 (32.4)	385 (30.3)	1725 (28.6)	1813 (30.1)
Not Performed	401 (44.7)	555 (43.7)	2844 (47.2)	2665 (44.3)
**Form** (%)				
Pulmonary	763 (85.0)	1079 (85.0)	5337 (88.6)	5143 (85.4)
Extrapulmonary	108 (12.0)	159 (12.5)	596 (9.9)	738 (12.3)
Both P & E	27 (3.0)	31 (2.4)	88 (1.5)	140 (2.3)
**Throat Culture** (%)				
Positive	79 (8.8)	111 (8.7)	587 (9.7)	508 (8.4)
Negative	88 (9.8)	83 (6.5)	571 (9.5)	399 (6.6)
In Progress	40 (4.5)	74 (5.8)	450 (7.5)	330 (5.5)
Not Performed	691 (76.9)	1001 (78.9)	4413 (73.3)	4784 (79.5)
**Tuberculin Skin Test** (%)				
No Reaction	61 (6.8)	79 (6.2)	414 (6.9)	366 (6.1)

Some Reaction	24 (2.7)	29 (2.3)	73 (1.2)	136 (2.3)
Strong Reaction	160 (17.8)	244 (19.2)	1198 (19.9)	1104 (18.3)
Not Performed	653 (72.7)	917 (72.3)	4336 (72.0)	4415 (73.3)
**Directly Observed Treatment** (%)				
DOT	434 (48.3)	692 (54.5)	3595 (59.7)	3198 (53.1)
No DOT	458 (51.0)	563 (44.4)	2417 (40.1)	2768 (46.0)
Unknown	6 (0.7)	14 (1.1)	9 (0.1)	55 (0.9)
**Rifampicin** = Not Taking (%)	20 (2.2)	21 (1.7)	202 (3.4)	102 (1.7)
**Isoniazid** = Not Taking (%)	18 (2.0)	17 (1.3)	193 (3.2)	81 (1.3)
**Ethambutol** = Not Taking (%)	245 (27.3)	348 (27.4)	1439 (23.9)	1689 (28.1)
**Streptomycin** = Not Taking (%)	889 (99.0)	1260 (99.3)	5990 (99.5)	5975 (99.2)
**Pyrazinamide** = Not Taking (%)	26 (2.9)	30 (2.4)	320 (5.3)	148 (2.5)
**Ethionamide** = Not Taking (%)	882 (98.2)	1253 (98.7)	5991 (99.5)	5944 (98.7)
**Other Drugs** = Not Taking (%)	861 (95.9)	1234 (97.2)	5765 (95.7)	5842 (97.0)
**AIDS** = No AIDS (%)	819 (91.2)	1207 (95.1)	5778 (96.0)	5705 (94.8)
**Alcoholism** = No Alcoholism (%)	809 (90.1)	1139 (89.8)	5451 (90.5)	5387 (89.5)
**Diabetes** = No Diabetes (%)	831 (92.5)	1183 (93.2)	5502 (91.4)	5614 (93.2)
**HIV** (%)				
Positive	89 (9.9)	69 (5.4)	261 (4.3)	354 (5.9)
Negative	526 (58.6)	744 (58.6)	3966 (65.9)	3507 (58.2)
In Progress	40 (4.5)	84 (6.6)	205 (3.4)	376 (6.2)

Not Undertaken	243 (27.1)	372 (29.3)	1589 (26.4)	1784 (29.6)
**Mental Disorder** = No Mental Disorder (%)	875 (97.4)	1260 (99.3)	5991 (99.5)	5975 (99.2)
**Other Disorder** = No Disorder (%)	777 (86.5)	1115 (87.9)	5379 (89.3)	5292 (87.9)
**Food Expenditure** (mean (sd))	209.50 (131.61)	184.69 (118.13)	192.67 (114.22)	183.22 (117.68)
**Energy Expenditure** (mean (sd))	40.17 (34.37)	32.45 (30.64)	36.22 (30.73)	32.70 (30.38)
**Gas Expenditure** (mean (sd))	33.69 (20.21)	32.75 (13.26)	33.04 (11.53)	32.86 (13.15)
**Water Expenditure** (mean (sd))	22.33 (20.15)	18.28 (21.99)	18.83 (18.93)	18.52 (20.43)
**Child Work =** No Child Worker (%)	881 (98.1)	1223 (96.4)	5893 (97.9)	5814 (96.6)
**Institutionalised** (%)				
None	820 (91.3)	1181 (93.1)	5688 (94.5)	5619 (93.3)
Military	30 (3.3)	33 (2.6)	173 (2.9)	172 (2.9)
Asylum	0 (0.0)	1 (0.1)	0 (0.0)	0 (0.0)
Orphanage	0 (0.0)	2 (0.2)	0 (0.0)	0 (0.0)
Psychiatric	1 (0.1)	1 (0.1)	0 (0.0)	0 (0.0)
Other	22 (2.4)	27 (2.1)	67 (1.1)	113 (1.9)
Unknown	25 (2.8)	24 (1.9)	93 (1.5)	117 (1.9)
**Work Acquired TB** (%)				
Got at work	20 (2.2)	24 (1.9)	109 (1.8)	114 (1.9)
Not at work	790 (88.0)	1134 (89.4)	5281 (87.7)	5370 (89.2)
Unknown	88 (9.8)	111 (8.7)	631 (10.5)	537 (8.9)

**State** (%)				
Rondonia	3 (0.3)	7 (0.6)	0 (0.0)	0 (0.0)
Acre	6 (0.7)	14 (1.1)	47 (0.8)	46 (0.8)
Amazonias	47 (5.2)	112 (8.8)	514 (8.5)	528 (8.8)
Roraima	2 (0.2)	5 (0.4)	1 (0.0)	15 (0.2)
Para	22 (2.4)	39 (3.1)	304 (5.0)	187 (3.1)
Amapa	0 (0.0)	2 (0.2)	0 (0.0)	0 (0.0)
Tocantins	7 (0.8)	3 (0.2)	15 (0.2)	16 (0.3)
Maranhao	48 (5.3)	70 (5.5)	288 (4.8)	353 (5.9)
Piaui	19 (2.1)	32 (2.5)	76 (1.3)	122 (2.0)
Ceara	118 (13.1)	214 (16.9)	1267 (21.0)	1040 (17.3)
Rio Grande do Norte	7 (0.8)	20 (1.6)	17 (0.3)	72 (1.2)
Paraiba	7 (0.8)	23 (1.8)	62 (1.0)	118 (2.0)
Pernambuco	27 (3.0)	63 (5.0)	128 (2.1)	287 (4.8)
Alagoas	18 (2.0)	35 (2.8)	294 (4.9)	166 (2.8)
Sergipe	15 (1.7)	36 (2.8)	150 (2.5)	166 (2.8)
Bahia	68 (7.6)	106 (8.4)	387 (6.4)	504 (8.4)
Minas Gerais	60 (6.7)	81 (6.4)	436 (7.2)	404 (6.7)
Espirito Santo	39 (4.3)	34 (2.7)	126 (2.1)	158 (2.6)
Rio de Janeiro	55 (6.1)	110 (8.7)	375 (6.2)	536 (8.9)
Parana	102 (11.4)	82 (6.5)	485 (8.1)	420 (7.0)

Santa Caterina	53 (5.9)	18 (1.4)	62 (1.0)	91 (1.5)
Rio Grande do Sul	117 (13.0)	115 (9.1)	758 (12.6)	588 (9.8)
Mato Grosso do Sul	18 (2.0)	16 (1.3)	63 (1.0)	68 (1.1)
Mato Grosso	17 (1.9)	20 (1.6)	116 (1.9)	80 (1.3)
Goias	14 (1.6)	9 (0.7)	24 (0.4)	41 (0.7)
Distrito Federal	9 (1.0)	3 (0.2)	26 (0.4)	15 (0.2)
**Income** (mean (sd))	197.39 (465.17)	65.22 (56.04)	74.05 (57.81)	68.36 (58.05)

Covariates are grouped by DAG node including *Sex & Age* (Sex, Age), *Race* (Race, Indigenous, Quilombola), *Education* (Education Level, Literacy), *Local Area* (Urban, Running Water, Sewage, Electricity, Water Store, Trash), *Type of TB* (Thorax X-Ray, Initial Bacilloscopy, Form, Throat Culture, Tuberculin Skin Test), *Directly Observed Treatment* (DOT), *Drugs* (Rifampicin, Isoniazid, Ethambutol, Streptomycin, Pyrazinamide, Ethionamide, Other Drugs), *Comorbidities* (AIDS, Alcoholism, Diabetes, HIV, Mental Disorder, Other Disorder), *Expenditure* (on Food, Energy, Gas, and Water), *Social Vulnerability* (Child Worker, Institutionalised, Work Acquired TB), *State*, and *Income.* Where duplicate variables existed, SINAN was used preferentially.

Appendix 2 – Missing Data

Though it was not the primary analytical method for this work, a confirmatory sensitivity analysis using multiple imputation was undertaken using the MICE (multiple imputation by chained equations) approach, as implemented in the MICE package in R.[1-2] The MICE package defaults of predictive mean matching and polytomous regression were used as the imputation methods for numeric and categorical variables respectively, creating 5 multiply imputed datasets.

The literature is still unclear as to whether the pooling of propensity scores themselves or the pooling of treatment estimates is the better approach after multiple imputation. Here, we followed Leyrat et al. (2016) and applied Rubin's rules to pool the ATT estimates from each imputed dataset.[3-4] The ATT estimates were based on a comparison between groups that were matched on the propensity score estimated by the same model specification used for Model A. The resulting estimated ATT was 7.22, in broad agreement with other results.

An approach combining multiple imputation and propensity score methods was not used for the primary analysis due to numerous unresolved questions that admit the possibility for an unknown amount of bias with regards to the estimation of variance after pooling, the timing of pooling datasets, the best number of datasets to impute, the best method for handling imputations, at a minimum. Practical guidelines for methods that more efficiently and robustly account for the incompleteness of data within estimation methods based on the propensity score are needed, but research in this area is ongoing.

References

1. Buuren S van, Groothuis-Oudshoorn K. mice: Multivariate Imputation by Chained Equations in R. J Stat Softw. 2011;45. Available: http://www.jstatsoft.org/v45/i03
2. Azur MJ, Stuart EA, Frangakis C, Leaf PJ. Multiple Imputation by Chained Equations: What is it and how does it work? Int J Methods Psychiatr Res. 2011;20: 40–49. doi:10.1002/mpr.329
3. Leyrat C, Seaman SR, White IR, Douglas I, Smeeth L, Kim J, et al. Propensity score analysis with partially observed confounders: how should multiple imputation be used? ArXiv160805606 Stat. 2016; Available: http://arxiv.org/abs/1608.05606
4. Rubin DB. Inference and missing data. Biometrika. 1976;63: 581–592. doi:10.1093/biomet/63.3.581
